# A Newly Developed Robot Suit Hybrid Assistive Limb Facilitated Walking Rehabilitation after Spinal Surgery for Thoracic Ossification of the Posterior Longitudinal Ligament: A Case Report

**DOI:** 10.1155/2013/621405

**Published:** 2013-11-28

**Authors:** Harutoshi Sakakima, Kosei Ijiri, Fumiyo Matsuda, Hiroyuki Tominaga, Takanori Biwa, Kazunori Yone, Yoshiyuki Sankai

**Affiliations:** ^1^Course of Physical Therapy, School of Health Sciences, Faculty of Medicine, Kagoshima University, 8-35-1 Sakuragaoka, Kagoshima 890-8544, Japan; ^2^Department of Orthopedic Surgery, Faculty of Medicine, Kagoshima University, Japan; ^3^Fujiyoshi Orthopedic Hospital, Kagoshima, Japan; ^4^Faculty of Systems and Information Engineering, Center for Cybernics Research, University of Tsukuba, Ibaraki 305-8575, Japan

## Abstract

Most patients with thoracic ossification of the posterior longitudinal ligament (OPLL) exhibit delayed recovery of gait dysfunction after spinal injury. The hybrid assistive limb (HAL) is a new robot suit controlling knee and hip joint motion by detecting very weak bioelectric signals on the surface of the skin. This study is to report the feasibility and benefits of patient-assistive HAL walking rehabilitation for facilitating locomotor function after spinal surgery. The patient was a 60-year-old woman with thoracic OPLL, and her motor and sensory paralyses did not improve after spinal surgery, indicating severe impairment in the paretic legs. The subject underwent 6 HAL sessions per week for 8 weeks, consisting of a standing and sitting exercise and walking on the ground with HAL. Clinical outcomes were evaluated before and after HAL training and 1 year after surgery. The subject improved considerably as a result of HAL training. Subsequently, her walking ability recovered rapidly, and she was able to walk unaided six months after surgery. This case study suggests that HAL training is a feasible and effective option to facilitating locomotor function and the early HAL training with physiotherapy may enhance motor recovery of patients with residual paralysis after surgery.

## 1. Introduction

Decompression is the primary treatment for patients with compressive myelopathy due to thoracic ossification of the posterior longitudinal ligament (OPLL) and ossification of the ligamentum flavum (OLF), but surgical outcomes vary. Studies of postoperative clinical outcomes of thoracic OPLL indicate that most patients exhibit delayed recovery of motor weakness in the lower limbs and gait dysfunction after surgery [1, 2]. Gait dysfunction is the most important negative surgical outcome, being a clinical deficit of spinal myelopathy [3].

**Figure 1:**
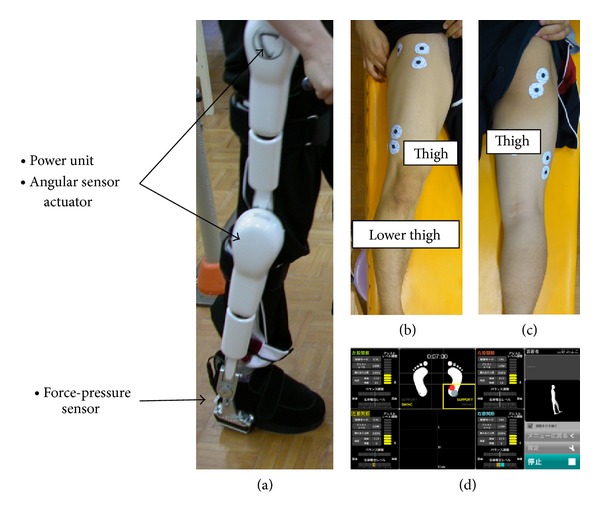
Newly-developed wearable robot suit, hybrid assistive limb (HAL). The HAL suit has power units and force-pressure sensors in the shoes. The power units consist of angular sensors and actuators on bilateral hip and knee joints (a). Muscle action potentials are detected through the electrodes on the anterior and posterior surface of the thigh ((b), (c)). Assist levels and force-pressure are shown on a computer monitor (d).

Robotic therapy is becoming increasingly common for gait rehabilitation after stroke or spinal cord injury, using an exoskeleton robotic device (e.g., Lokomat, LOPES exoskeleton robot) or a robotic device with foot-driven plates (e.g., Gait Trainer GT I, Haptic Walker) [4–6]. The robot suit hybrid assistive limb (HAL) is a new robot suit to assist voluntary control of knee and hip joint motion by detecting very weak bioelectric signals on the surface of the skin [7]. The HAL suit is a hybrid control system comprising cybernic voluntary control (CVC) and cybernic autonomous control (CAC) subsystems and has power units and force-pressure sensors in the shoes [8, 9]. The power units consist of angular sensors and actuators on the bilateral hip and knee joints (Figure 1). The HAL suit can support the wearer's motion by adjusting the level and timing of assistance [7]. HAL training, using muscle activity, has the potential to intensify the feedback by evoking by an appropriate motion more strongly than standard robot training [9]. HAL training has been shown to improve gait speed or cadence for chronic stroke and incomplete spinal cord injury [8, 9]. However, no studies have attempted to clarify the feasibility of rehabilitation with HAL for patients with residual paralysis after spinal decompression for thoracic OPLL or OLF.

This case was markedly improved locomotor function by training with HAL, although recovery did not start until 7 weeks after spinal decompression of thoracic OPLL. Therefore, we report a case of patient-assistive HAL walking rehabilitation from an early stage for facilitating locomotor functions for patients with severe residual paralysis.

## 2. Case Presentation

**Figure 2:**
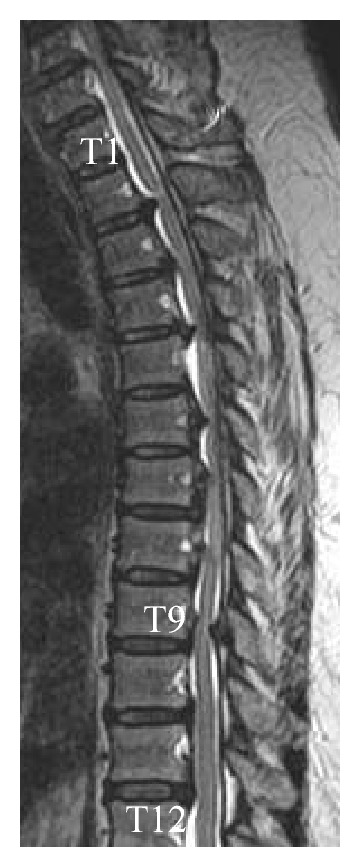
T1-weighted magnetic resonance imaging showed areas of OPLL extending from T2 to T8 and T9/T10 OYL.

A 60-year-old woman (body mass index: 31.1 kg/m^2^) presented with onset of pain and numbness in her right lower limb and gait disturbance. The diagnosis was cervico-thoracic OPLL. After 15 months, her symptoms had gradually progressed, showing motor and sensory paresis of the lower limb and urinary disturbance. Magnetic resonance imaging showed areas of OPLL extending from T2 to T8 and T9/T10 OYL (Figure 2). Because of progressive myelopathy, she underwent posterior decompression surgery two times. However, she showed aggravation of myelopathy after the second surgery, complete motor and sensory paralysis below T4, and urinary retention. She then underwent anterior decompression surgery to remove the OPLL. Active movement of her toes was weak at 1 day after surgery. She underwent physical therapy (PT) with pharmacological and high atmospheric pressure oxygen inhalation therapy. However, her motor and sensory paralyses did not improve. She was still bedridden 7 weeks after surgery and at risk of disuse syndrome. We decided to use HAL in addition to the conventional PT such as muscle strength exercises and range of motion exercises. Before participating in walking exercise using HAL, the subject provided informed consent, and the study was approved by the Ethics Committee of the Kagoshima University Faculty of Medicine.

Clinical assessments were carried out at the initial evaluation (at 7 weeks after final surgery) and 8 weeks and 8 months after HAL intervention (15 weeks and 1 year after surgery, resp., Table 1). After the initial evaluation, the subject underwent 6 HAL sessions of 70 minutes per week for 8 weeks. Sessions consisted of a standing and sitting exercise, and walking on the ground with HAL. Standing and walking training started in parallel bars with HAL. A typical 70-minute HAL training session proceeded as follows: preparation of electrodes, putting on the HAL suit, and computer setup (15 min); HAL training (40 min, including rest time); taking off the HAL suit and electrodes (15 min). Three therapists implemented the training. The HAL suit has a hybrid control system comprising the CVC and CAC. The CVC mode of the HAL suit can support the patient's voluntary motion according to the voluntary muscle activity and the assistive torque provided to each joint [9]. This study used the CVC mode, which allows the operator to adjust the degree of physical support to the patient's comfort and gradually reduce support as training progresses. After the end of HAL intervention, the patient underwent conventional PT without HAL in another hospital, and she was discharged 10 months after surgery.

**Figure 3:**
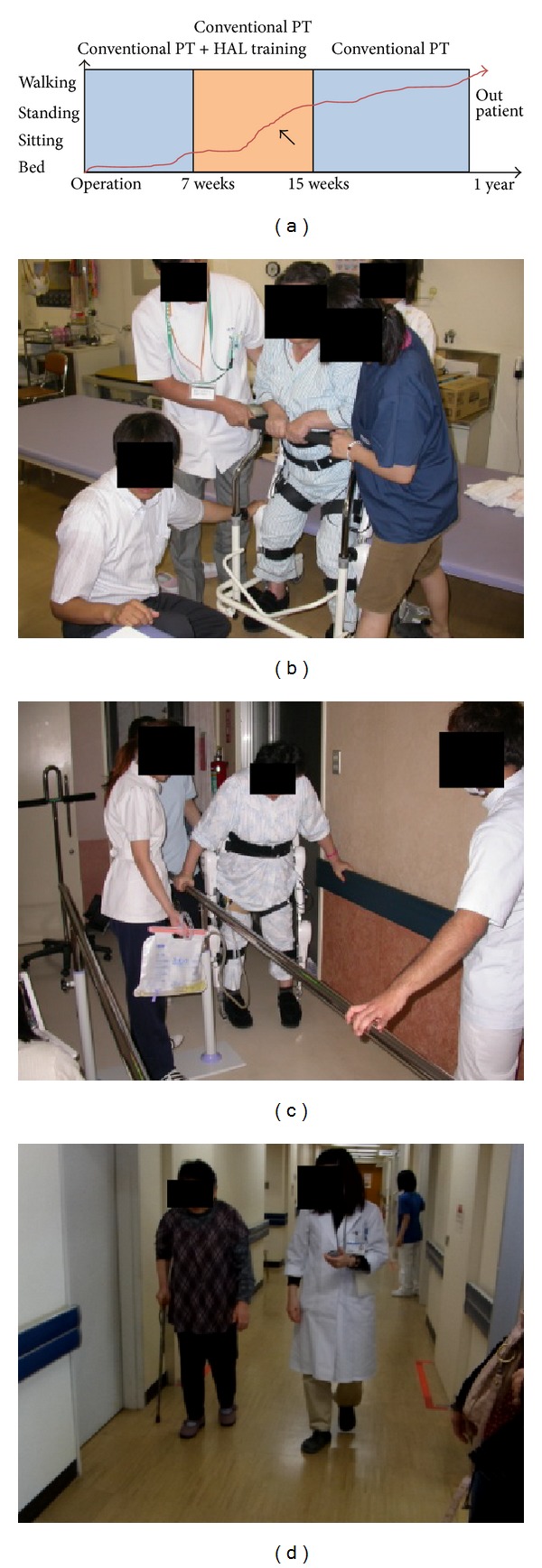
Improvement time course of activity of the patient in a schematic view (a). Although she underwent conventional physical therapy (PT), she was still bedridden 7 weeks after surgery. Locomotor functions of the patient improved considerably by intervention of the robot suit hybrid assistive limb (HAL) training. Subsequently, the walking ability recovered rapidly (arrow). When she put on the HAL at baseline, she could stand for only few seconds with assistance from three tree therapists (b). However, she could walk in the parallel bars at 12 weeks after surgery (c) and could walk independently 1 year after surgery (d).

Locomotor functions of the patient improved considerably by the intervention of HAL training. Subsequently, her walking ability recovered rapidly and she was able to walk independently six months after surgery.  Figure 3 shows the improvement time course of activity of the patient in a schematic view. At 15 weeks after surgery, she was able to sit without back support and transfer to a wheelchair independently. She could walk in parallel bars without HAL, although rocking of the knee was observed while standing. At 1 year after surgery, she was able to walk independently with a T-cane.

## 3. Discussion

This case report describes the feasibility of facilitating locomotor functions with HAL training for patients with residual paralysis after spinal surgery. Matsumoto et al. [10] reported improvement in 36.8% of patients but deterioration in 8.4% after spinal surgery for thoracic OPLL in a retrospective multicenter study of 154 Japanese hospitals. The present patient was operated on 3 times and showed aggravation of her lower limb myelopathy after surgery. Although recovery did not start until 7 weeks after surgery, her locomotor function markedly improved by combining training with HAL, suggesting that HAL training facilitated recovery of locomotor functions. The HAL may facilitate rehabilitation by providing postural support and assisted voluntary muscle activity during ambulation.

HAL is a robotic device with potential rehabilitation applications that are dependent on the physical support it can provide [9]. A patient's recovery of locomotor functions may be due to changes in plasticity of the spinal cord and supraspinal centers. Appropriate sensory inputs, such as maximum weight loading, facilitating proper trunk posture, and hip extension, are essential for maximizing functional recovery [11]. Sensory input evoked HAL-induced motion may affect the central nervous system, resulting in recovery of locomotor functions. Furthermore, the visual feedback of watching a display indicating the center of gravity and range of motion of the lower limbs may also affect the central nervous system. HAL rehabilitation can be implemented safely and effectively for early mobilization and gait training for patients with residual paralysis after spinal surgery.

This study had a clear limitation in that the HAL training was started relatively soon after surgery. However, even if this patient was still in the recovery period, her locomotor function markedly improved by combining training with HAL. HAL training at an early stage may be necessary to prevent disuse syndrome such as muscle weakness in the lower limbs or joint contracture. The subject may also have experienced improved motivation for rehabilitation by HAL training use from an early stage, because she had been bedridden for 7 weeks after surgery. The findings from this case report suggest that HAL training for voluntary control of leg joint motion from an early phase is a safe and effective option for restoring locomotor functions in patients with residual paralysis after spinal surgery.

## 4. Conclusion

We concluded that for patients of thoracic OPLL, the early HAL training with physiotherapy may enhance motor recovery after surgery. Early mobilization using HAL may be also advocated to prevent post surgery complications, such as contractures and deep vein thrombosis.

## Figures and Tables

**Table 1:** Baseline and clinical assessment during follow-up period.

	7 weeks (baseline)	15 weeks (end of HAL)	After 1 year
MMT (U/L)	5/1-2	5/3-4	5/4^+^-5
JOA score	8	11	13
ASIA classification	C	D	D
ASIA score (lower limbs)	23	34	42
WISCI II	0	8	20
FIM motor score	22	40	83

MMT: Manual muscle testing. JOA: Japan orthopedic association (maximum score: 17). ASIA: American spinal injury association. WISCI: Walking index for spinal cord injury (score range 0 to 20). FIM: Functional independence measure (maximum score: 91).
